# Awareness and use of flavor accessories for combustible tobacco products: A 2024 cross-sectional survey of high school students in Connecticut, USA

**DOI:** 10.1371/journal.pone.0341327

**Published:** 2026-02-18

**Authors:** Christina N. Kyriakos, Krysten W. Bold, Meghan E. Morean, Suchitra Krishnan-Sarin, Danielle R. Davis, Grace Kong

**Affiliations:** Department of Psychiatry, Yale School of Medicine, Yale University, New Haven, Connecticut, United States of America; Newcastle University, UNITED KINGDOM OF GREAT BRITAIN AND NORTHERN IRELAND

## Abstract

**Significance:**

Flavor accessories (e.g., flavor capsules), which are separate products that can be used with combustible tobacco products to alter their flavor, are on the market. These new products may bypass flavor restrictions and appeal to youth, yet no research has examined US youth awareness or use of flavor accessories. This study aimed to examine awareness and use of flavor accessories among a sample of youth in the US.

**Methods:**

A school-based survey of 4,760 Connecticut high school students (mean age = 15.9, SD = 1.2) was conducted in April-May 2024. All youth reported on awareness of flavor accessories (i.e., flavor capsules, sprays/drops, cards). Youth who had ever used combustible tobacco products (i.e., cigarettes, cigars, cigarillos) or cannabis blunts, which are all products that can be used with flavor accessories (N = 868), also reported on ever use of flavor accessories. Differences in awareness by ever product user type (i.e., exclusive combustible tobacco (n = 173), exclusive blunt (n = 461), dual tobacco and blunt (n = 234)) were assessed using Bonferroni-corrected chi-square tests.

**Results:**

Overall, 22.9% of youth were aware of at least one type of flavor accessory. Among ever users of combustible tobacco or blunts, awareness of at least one type of flavor accessory was 32.8%, with awareness most common for flavor capsules (19.6%), followed by sprays/drops (16.3%), and cards (9.5%). Among this group, more dual users of combustible tobacco and blunts were aware of at least one type of flavor accessory compared to exclusive users of combustible tobacco (44.1% vs 26.3%, p = 0.002) and compared to exclusive users of blunts (44.1% vs 30.8%, p = 0.005). Among ever users of combustible tobacco or blunts, ever use of any flavor accessory was 7.6%, ranging from 4.7% for flavor capsules, 3.5% for sprays/drops, and 2.7% for cards.

**Conclusions:**

Nearly one-quarter of a sample of Connecticut high school youth were aware of flavor accessories, with differences in awareness by product user groups, although ever use of these products was lower. Continued monitoring of flavor accessories is critical for informing regulatory actions and interventions.

## Introduction

Combustible tobacco use remains the leading cause of preventable death in the United States (US), attributable to approximately 480,000 deaths annually, and disease in nearly every organ of the body [1]. Prevention efforts focused on youth are essential for reducing morbidity and mortality. Flavors play a central role in youth initiation and therefore should be a key focus of these efforts [2–4]. Internal industry documents reveal that flavors are intentionally used to recruit young people to use tobacco products [5,6]. By enhancing the palatability and reducing the harshness of tobacco, flavors increase the appeal of tobacco products, which can lead to experimentation, particularly among youth [7,8]. Furthermore, initiating tobacco use with flavored tobacco products is associated with an increased likelihood of continued use among youth [3].

Flavor accessories, which are separate products that can add flavor to combustible tobacco products, have recently emerged in the global market [9–11]. These products come in a variety of forms, such as capsules, cards, and sprays, and are offered in a myriad of flavors [9,12–14]. For instance, flavor capsules are gelatin balls filled with flavoring liquid that can be inserted into a cigarette filter and crushed by the user to release flavor, essentially creating a ‘do-it-yourself’ flavor capsule cigarette [9,14]. A study that conducted an inventory of different types of flavor accessories across eight countries, including the US, identified nine distinct product types (i.e., capsules, cards, filter tips and tubes, drops, sprays, rolling paper, aroma markers, flavor stone, and flavor powder) and 118 unique flavors [9]. With the role of flavors in enhancing the appeal of tobacco and in facilitating smoking initiation particularly among youth, there is a need to understand the extent to which flavor accessories are known and used among this population.

In addition to increasing tobacco product attractiveness, flavor accessories may also undermine restrictions on flavors in combustible tobacco products, particularly when these policies do not explicitly include these accessories. In some countries, the introduction of flavor accessories coincided with implementation of menthol cigarette bans that did not cover these products, creating a loophole that enabled them to be marketed as alternatives to menthol cigarettes [9,11,12]. In the US, federal law prohibits characterizing flavors in cigarettes, but exempts menthol and does not address flavor accessories. Although a proposed rule to restrict the sale of menthol cigarettes, including accessories, was introduced, it was withdrawn in January 2025 [15]. As a result, flavor accessories remain unregulated at the US national level and may further circumvent sub-national menthol restrictions that do not explicitly include them.

Beyond their compatibility with various combustible tobacco products, flavor accessories may also be used with combustible cannabis products, such as blunts. Studies to date have not yet examined how these flavor accessories are used for cannabis smoking. However, a focus group study showed that youth found sweet, fruity flavors to be appealing in creating and smoking blunts [16]. As dual use of tobacco and cannabis is common among young people, products that can be used to enhance the appeal of both substances warrant monitoring given that they may maintain or reinforce user behaviors, particularly in a restricted flavor environment [17,18].

Little is known about youth awareness and use of flavor accessories in the US, indicating a critical research gap. However, studies conducted in other countries suggest that flavor accessories are popular among youth and young adults [19–22]. For instance, in England and in Canada where menthol cigarettes are banned, but not accessories, 24.2% of youth who smoke in England and 15.6% in Canada reported past-30-day use of menthol accessories in 2021 [22]. Yet, to our knowledge, no studies have examined this among youth in the US. Therefore, this study aimed to examine awareness and use of flavor accessories among a sample of youth in the US. We further aimed to assess awareness of flavor accessories by different types of combustible tobacco and cannabis product user groups. Understanding youth engagement with these emerging products can inform effective regulatory efforts and interventions.

## Materials and methods

### Study design

A cross-sectional, school-based survey was conducted in a convenience sample of students in eight Connecticut high schools between April 10, 2024 and May 23, 2024 (N = 4,760). Schools were selected to represent diverse socioeconomic backgrounds using District Reference Groups to be reflective of the Connecticut population and included a mix of mid-sized and large schools located in both suburban and urban communities. Online surveys were administered by teachers during school hours. Teachers read standardized instructions to students using a script provided by the research team. Surveys took approximately 20 minutes to complete. The survey was iteratively developed by a team of tobacco researchers, using validated measures where applicable, as part of an ongoing study of biannual surveys conducted in Connecticut schools since 2013. The study was approved by the Yale University Institutional Review Board (#1207010580) and by all participating schools. Parents were informed of the study through an e-mail sent out by the school and could opt-out their child from participation (n = 29). The Yale University Institutional Review Board issued a waiver of written assent/consent to maintain participant anonymity. Thus, completion of the survey was considered consent for students ages 18 years and older or assent for students younger than 18 years.

### Measures

#### Sociodemographics.

Participants reported their age (13−19 years), grade (9^th^-12^th^), sex at birth (Female, Male), perception of their family’s overall financial situation (we do not meet basic expenses, we just meet basic expenses, we meet expenses with a little left over, we live comfortably) [23], ethnicity defined as Hispanic, Latino or Spanish origin (Yes, No), and Race (White, Black, Asian, American Indian or Alaska Native, Native Hawaiian or pacific Islander, Other).

### Ever use of combustible tobacco products or blunts. 

We assessed ever use of combustible tobacco products or blunts with the question “Have you ever tried…[product]?” (yes, no), for each: cigarette, large cigar (“like a traditional “Cuban” cigar, without marijuana”), cigarillo or little cigar (“short, narrow cigar such as “Black & Mild”, without marijuana”), blunt (“cigar, cigarillo, little cigar or blunt wrap filled with marijuana”). Each question was accompanied by an image and description. Those who reported ‘no’ for each product were considered ‘never users of combustible tobacco or blunts’. Those who had tried any of these products were considered ‘ever users of combustible tobacco products or blunts’. This group was further categorized as: (1) exclusive users of combustible tobacco (defined as cigarettes, large cigars, or cigarillos) with no blunt use, (2) exclusive users of blunts with no combustible tobacco use, and (3) dual users of blunts and combustible tobacco use.

### Awareness and ever use of flavor accessories. 

The survey included descriptions and images of three types of flavor accessories: (1) Flavor capsules, crush balls, and flavor popping beads (“Small-prefilled beads that you buy separately from tobacco products. You push them into a cigarette, cigarillo, or cigar and crush them”); (2) Tobacco flavoring sprays and drops (“Come in many flavors (e.g., fruit, menthol, mint) and are put directly onto loose tobacco, the filters of cigarettes, or onto cigarettes, cigarillos, and cigars to add flavor”); and (3) Tobacco flavoring cards (“Small paper cards that are about the size of a pack of cigarettes. They come in many flavors and are slid into a pack of cigarettes or other tobacco products”).

Awareness of each flavor accessory was assessed among all participants with the question, “Before today, had you ever heard of [product]” (yes, no). Ever use of each product was assessed among adolescents who reported awareness of the product and who reported ever use of combustible tobacco or blunts, with the question “Have you ever used [product]?” (yes, no, don’t know). ‘Don’t know’ responses were coded as never using the product. Those who reported ever use of combustible tobacco or blunts, but reported that they had never heard of a given flavor accessory product were also coded as never using the product.

Two composite variables reflecting awareness of ‘at least one type of flavor accessory’ and use of ‘at least one type of flavor accessory’ were created where responses were coded as ‘yes’ if they indicated awareness or use of at least one of the flavor accessories, respectively.

### Analysis

To examine awareness of flavor accessories, we ran descriptive statistics for each type of flavor accessory and the composite variable of ‘at least one type of flavor accessory’ across different user groups: (1) overall sample, (2) never users of combustible tobacco or blunts, and (3) ever users of combustible tobacco or blunts. Further, we ran descriptive statistics for the three ever product user groups (i.e., exclusive combustible tobacco, exclusive blunt, and dual tobacco and blunt) and assessed differences in awareness of flavor accessories across groups using Bonferroni-corrected chi-square tests. The Bonferroni-corrected significance threshold was set at p < 0.017 (α = 0.05/3).

To examine ever use of flavor accessories, we ran descriptive statistics among ever users of combustible tobacco or blunts and the three ever product user groups. Given the small samples sizes (S1 Table), chi-square comparisons were not conducted to compare differences in ever use of flavor accessories across ever product user groups.

All analyses were conducted using complete case data, with percentages and comparisons calculated only among participants with available responses for each outcome. To assess the impact of missing data, we conducted a sensitivity analysis in which all missing responses for flavor accessory awareness and ever use were recoded as “no” (i.e., not aware or never used). This approach assumes that participants who did not provide a response were unaware of or had not used the product. While generally consistent with the main findings, percentages were lower compared to the complete case analysis, given the conservative assumption that missing responses indicated lack of awareness or use (S2 Table).

All analyses were conducted in Stata/MP 18.0.

## Results

Sample characteristics are presented for the overall sample (N = 4,760) and among the sub-sample of ever users of combustible tobacco or blunts (N = 868) (Table 1).

**Table 1:** Sample characteristics among Connecticut high school youth, 2024.

	Overall sample (N = 4,760)			Ever users of combustible tobacco or blunts (N = 868)		
	n	%	95%CI	n	%	95%CI
**Age** Mean (SD)		15.9 (1.24)			16.2 (1.26)	
**Grade** ^**1**^						
9th	1370	28.9	(27.6, 30.2)	168	19.4	(16.9, 22.2)
10th	1423	30.0	(28.7, 31.3)	209	24.1	(21.4, 27.1)
11th	1137	24.0	(22.8, 25.2)	254	29.2	(26.3, 32.3)
12th	811	17.1	(16.1, 18.2)	236	27.2	(24.4, 30.3)
**Sex at birth** ^**1**^						
Female	2438	51.4	(50.0, 52.8)	432	49.9	(46.5, 53.2)
Male	2303	48.6	(47.1, 50.0)	434	50.1	(46.8, 53.4)
**Perception of family’s overall financial situation** ^**1**^						
Do not meet basic expenses	135	2.9	(2.4, 3.4)	48	5.6	(4.2, 7.3)
Just meet basic expenses	1053	22.3	(21.1, 23.5)	226	26.2	(23.3, 29.2)
Meet expenses with a little left over	1253	26.5	(25.3, 27.8)	257	29.8	(26.8, 32.9)
Live comfortably	2278	48.3	(46.8, 49.7)	332	38.5	(35.3, 41.8)
**Hispanic, Latino, or Spanish origin** ^**1**^						
Yes	1804	38.1	(36.7, 39.5)	370	42.7	(39.5, 46.0)
No	2931	61.9	(60.5, 63.3)	496	57.3	(53.9, 60.5)
**Race** (select all that apply)						
White	2842	60.9	(59.4, 62.2)	494	57.4	(54.1, 60.7)
Black	1047	22.4	(21.2, 23.6)	265	30.8	(27.8, 34.0)
Asian	310	6.6	(6.0, 7.4)	46	5.3	(4.0, 7.1)
American Indian/ Alaska Native	137	2.9	(2.5, 3.5)	36	41.9	(3.0, 5.7)
Pacific Islander/ Native Hawaiian	61	1.3	(1.0, 1.7)	16	1.9	(1.1, 3.0)
Other race	230	4.9	(4.3, 5.6)	43	5.0	(3.7, 6.7)
**Product ever use** (select all that apply)						
Cigarettes	299	6.3	(5.6, 7.1)	299	34.4	(31.3, 37.7)
Cigars	179	3.8	(3.3, 4.4)	179	20.6	(18.1, 23.4)
Cigarillos	128	2.7	(2.3, 3.2)	128	14.7	(12.5, 17.3)
Blunt	695	14.7	(13.7, 15.8)	695	80.1	(77.3, 82.6)

^1^Values are rounded to the nearest tenth. Unrounded values sum to 100%.

### Awareness of flavor accessories

We found that 22.9% (95%CI: 21.6–24.2%) of the overall sample (N = 4,760) were aware of at least one type of flavor accessory, with awareness highest for flavor capsules (13.0%), followed by flavor sprays or drops (12.8%), and flavoring cards (7.3%). Among ever users of combustible tobacco or bunts (N = 868), 32.8% (95%CI: 29.2–36.6%) were aware of at least one type of flavor accessory, with awareness also highest for flavor capsules (19.6%), followed by flavor sprays or drops (16.3%), and flavoring cards (9.5%). Among never users of combustible tobacco or blunts, (N = 3,851), 21.0% (95%CI: 19.7–22.4%) were aware of at least one type of flavor accessory, with awareness most common for flavor sprays or drops (12.1%), followed by flavor capsules (11.8%), and flavoring cards (6.9%) (Table 2).

**Table 2:** Awareness of flavor accessories by product user groups among Connecticut high school youth, 2024.

	At least one type of flavor accessory			Flavor capsules, crush balls, popping beads			Flavoring sprays/ drops			Flavoring cards		
User Group	n	%	95%CI	n	%	95%CI	n	%	95%CI	n	%	95%CI
Overall sample (N = 4,760)	921	22.9	(21.6, 24.2)	527	13.0	(12.0, 14.1)	516	12.8	(11.8, 13.8)	293	7.3	(6.5, 8.1)
Never users of combustible tobacco or blunts (N = 3,851)	714	21.0	(19.7, 22.4)	402	11.8	(10.7, 12.9)	413	12.1	(11.1, 13.3)	233	6.9	(6.1, 7.8)
Ever users of combustible tobacco or blunts (N = 868)	207	32.8	(29.2, 36.6)	125	19.6	(16.7, 22.9)	103	16.3	(13.6, 19.4)	60	9.5	(7.5, 12.1)
Exclusive combustible tobacco (N = 173)	35	26.3	(19.6, 34.3)	25	18.1	(12.5, 25.4)	11	8.0	(4.5, 13.8)	8	5.8	(2.9, 11.2)
Exclusive blunt (N = 461)	108	30.8	(26.1, 35.8)	59	16.6	(13.1, 20.9)	58	16.5	(13.0, 20.8)	24	6.9	(4.6, 10.0)
Dual (N = 234)	63	44.1	(36.1, 52.3)	41	28.5	(21.7, 36.4)	34	23.6	(17.4, 31.2)	28	19.6	(13.9, 26.9)

Combustible tobacco defined as cigarettes, large cigars, or cigarillos; Blunt defined as a cigar, cigarillo, little cigar or blunt wrap filled with marijuana.

‘Exclusive combustible tobacco’ = ever use of combustible tobacco and never use of blunts; ‘Exclusive blunt’ = ever use of blunts and never use of combustible tobacco; ‘Dual’ = ever use of combustible tobacco and ever use of blunts

See Table 3 for chi-square and Bonferroni-corrected pairwise comparison p-values between product user groups of combustible tobacco or blunts.

When examining awareness across different ever product user groups, we found that those who were dual users of combustible tobacco and blunts (N = 234) reported higher awareness of at least one type of flavor accessory compared to exclusive users of combustible tobacco (N = 173) (44.1% vs 26.3%, p = 0.002) and compared to exclusive users of blunts (N = 355) (44.1% vs 30.8%, p = 0.005). Awareness of flavor capsules was higher among dual users compared to exclusive blunt users (28.5% vs 16.6%, p = 0.003). Similarly, awareness of flavoring cards was higher among dual users compared to exclusive blunt users (19.6% vs 5.8%, p < 0.001), as well as compared to exclusive combustible tobacco users (19.6% vs 6.9%, p = 0.001). Awareness of flavoring sprays or drops was higher among dual users compared to exclusive combustible tobacco users (23.6% vs 8.0%, p = 0.014) and higher among exclusive blunt users compared to exclusive combustible tobacco users (16.5% vs 8.0% p < 0.001) (Table 2 and Table 3).

**Table 3:** Chi-square and pairwise comparison p-values for awareness of flavor accessories by product user groups among ever users of combustible tobacco or blunts (N = 868), Connecticut high school youth, 2024.

	At least one type of flavor accessory	Flavor capsules, crush balls, popping beads	Flavoring sprays/ drops	Flavoring cards
	p-value	p-value	p-value	p-value
Chi-square (overall)	**0.003**	**0.009**	**0.002**	**<0.001**
Exclusive combustible tobacco vs Exclusive blunt	0.328	0.692	**<0.001**	0.684
Exclusive combustible tobacco vs Dual	**0.002**	0.040	**0.014**	**0.001**
Exclusive blunt vs Dual	**0.005**	**0.003**	0.066	**<0.001**

Combustible tobacco defined as cigarettes, large cigars, or cigarillos; Blunt defined as a cigar, cigarillo, little cigar or blunt wrap filled with marijuana

‘Exclusive combustible tobacco’ = ever use of combustible tobacco and never use of blunts; ‘Exclusive blunt’ = ever use of blunts and never use of combustible tobacco; ‘Dual’ = ever use of combustible tobacco and ever use of blunts

**Bolded**= Statistically significant at Bonferroni-corrected threshold (p<0.017).

### Ever use of flavor accessories

Among youth who reported ever use of combustible tobacco or blunts (N = 868), 7.6% (95%CI: 5.8–10.0%) had ever used at least one type of flavor accessory. Ever use was highest for flavor capsules (4.7%, 95%CI: 3.3–6.7%), followed by flavoring sprays or drops (3.5%, 95%CI: 2.3–5.3%), and flavoring cards (2.7%, 95%CI: 1.7%−4.3%) (S1 Table).

## Discussion

To our knowledge, this is the first study to examine awareness and ever use of flavor accessories for combustible tobacco products in a sample of youth in the US. We found that nearly a quarter of high school students, including those who use combustible tobacco, blunts, and those who have never used these products in Connecticut were aware of at least one type of flavor accessory, with awareness higher among ever users of combustible tobacco or blunts compared to never users. Awareness was most common for flavor capsules, followed by sprays or drops, and cards. Awareness differed by ever product user groups, with dual users of combustible tobacco and blunts generally having the greatest awareness. Ever use of flavor accessories was less common. These findings are concerning because flavor accessories have the potential to increase the appeal of combustible tobacco and cannabis products through offering an enticing range of flavors and innovative design characteristics [21]. These products may further undermine flavor restrictions if not covered in policies.

Our finding that flavor capsules were the most familiar type of flavor accessory aligns with other studies [9,24]. For instance, a study using a standardized search of web shops across eight countries, including the US, found that flavor capsules were the most frequently reported flavor accessory on the market and had more brands and manufacturers than any of the eight other accessory types identified [9]. Moreover, menthol capsules were the most commonly reported menthol accessories ever used among a sample of Connecticut adults who smoke menthol cigarettes (29.3%), exceeding use of menthol filter tips (24.1%, cards (17.2%), and sprays (16.4%) [24]. This may partially be explained by the increasing promotion of flavor capsule accessories on platforms such as TikTok [14]. Social media can amplify exposure and susceptibility to tobacco use among young people [25]. In addition, flavor capsule accessories are designed to mimic flavor capsule cigarettes (products that contain a crushable capsule already embedded in the filter), which have gained market share in the US [26,27] and are known to be especially appealing to youth [28].

Although no prior studies have explored flavor accessories for cannabis blunts, our finding that dual users of combustible tobacco and blunts were more aware of flavor accessories may indicate that these products are gaining traction in the market, highlighting an area for future investigation. Indeed, one company sells filter tips pre-loaded with flavor capsules for use with blunts [29]. This company states on its website that “younger consumers, particularly those interested in unique experiences, flock to products that offer something different. ‘Squeeze and pop’ technology resonates well with this group, providing novelty and engagement” [29]. It is also possible that those who use multiple substances are more likely to be informed about a range of products available through their familiarity with various products or greater exposure to product advertising [30].

We also found flavor drops and sprays were more commonly known among exclusive users of blunts compared to exclusive users of combustible tobacco. While youth find flavors appealing in using blunts, [31] it is unknown how these flavor accessories are used in blunts. Research is needed to better understand the landscape of these products available for use across different types of tobacco and cannabis products.

This study is limited by its use of a convenience sample and restricted geographical location, which may limit the generalizability of findings. However, this sample of youth in Connecticut offered a uniquely beneficial opportunity for understanding the awareness and use of flavor accessories in a place where menthol cigarettes remain unrestricted. As such, the findings provide a valuable baseline assessment prior to potential future policy changes. Further, the small sample sizes of those who had ever used the examined products precluded additional analyses, such as assessing differences by sociodemographic characteristics, which remains an important area for further research. It should be noted that this study did not focus on examining flavor accessories that can be used with e-cigarettes, however such products (e.g., Puff Krush flavor capsules) [32] are also available.

Despite these limitations, this study provides novel data on US youth awareness and use of flavor accessories and offers important policy insights. Given that these products contain attributes and ingredients that may enhance the appeal, addictiveness, and toxicity of tobacco products, flavor restrictions may be most effective if they include flavor accessories, such as Massachusetts and California’s policies, which prohibit these products (referred to as ‘tobacco product flavor enhancers’) [33,34]. Moreover, closing regulatory gaps in online sales and marketing, such as by broadening the definition of tobacco products to encompass flavor accessories on social media platforms, may also be necessary to address loopholes [14]. Findings highlight the need for continued research to better understand how flavor accessories are being promoted and used.

## Conclusions

We found that nearly one-quarter of youth in a Connecticut high school sample were aware of flavor accessories, which are products that may increase the appeal of tobacco and circumvent tobacco product flavor restrictions. While ever use was relatively low, findings warrant continued monitoring.

## Supporting information

S1 Table Ever use of flavor accessories by product user groups among Connecticut high school youth who ever used combustible tobacco or blunts (N = 868), 2024. (DOCX)

S2 Table Sample sizes and sensitivity analyses treating missing responses as “No” (not aware/never used) among overall sample and ever users of combustible tobacco or blunts, Connecticut high school youth, 2024. (DOCX)

S1 File Data file. (XLSX)
