# Supplementary material for: Awareness and use of flavor accessories for combustible tobacco products: A 2024 cross-sectional survey of high school students in Connecticut, USA
Source: PLoS One. 2026 Feb 18;21(2):e0341327. doi: 10.1371/journal.pone.0341327 (PMC12915970; doi:10.1371/journal.pone.0341327)
Supplement: S1 Table — (DOCX) [file pone.0341327.s001.docx]

**S1 Table. Ever use of flavor accessories by product user groups among Connecticut high school youth who ever used combustible tobacco or blunts (N=868), 2024**

|  | **At least one type of flavor accessory** | | | **Flavor capsules, crush balls, popping beads** | | | **Flavoring sprays/ drops** | | | **Flavoring cards** | | |
| --- | --- | --- | --- | --- | --- | --- | --- | --- | --- | --- | --- | --- |
| **User Group** | **n** | **%** | **95%CI** | **n** | **%** | **95%CI** | **n** | **%** | **95%CI** | **n** | **%** | **95%CI** |
| Ever users of combustible tobacco or blunts (N=868) | 48 | 7.6 | (5.8, 10.0) | 30 | 4.7 | (3.3, 6.7) | 22 | 3.5 | (2.3, 5.3) | 17 | 2.7 | (1.7, 4.3) |
| Exclusive combustible tobacco (N=173) | 12 | 8.8 | (5.0, 14.8) | 9 | 6.5 | (3.4, 12.1) | 3 | 2.2 | (0.7, 6.5) | 1 | 0.7 | (0.1, 5.9) |
| Exclusive blunts (N=461) | 11 | 3.1 | (1.7, 5.6) | 4 | 1.1 | (0.4, 3.0) | 5 | 1.4 | (0.6, 3.4) | 5 | 1.4 | (0.6, 3.4) |
| Dual (N=234) | 25 | 17.5 | (12.1, 24.6) | 17 | 11.8 | (7.5, 18.2) | 14 | 9.7 | (5.8, 15.8) | 11 | 7.7 | (4.3, 13.4) |

Combustible tobacco defined as cigarettes, large cigars, or cigarillos; Blunt defined as a cigar, cigarillo, little cigar or blunt wrap filled with marijuana.

‘Exclusive combustible tobacco’= ever use of combustible tobacco and never use of blunts; ‘Exclusive blunt’= ever use of blunts and never use of combustible tobacco; ‘Dual’= ever use of combustible tobacco and ever use of blunts
