# Supplementary material for: Awareness and use of flavor accessories for combustible tobacco products: A 2024 cross-sectional survey of high school students in Connecticut, USA
Source: PLoS One. 2026 Feb 18;21(2):e0341327. doi: 10.1371/journal.pone.0341327 (PMC12915970; doi:10.1371/journal.pone.0341327)
Supplement: S2 Table — (DOCX) [file pone.0341327.s002.docx]

**S2 Table. Sample sizes and sensitivity analyses treating missing responses as “No” (not aware/never used) among overall sample and ever users of combustible tobacco or blunts, Connecticut high school youth, 2024**

| \|  \| **Overall sample (N=4760)** \| \| \| \| \| \| **Ever users of combustible tobacco or blunts (N=868)** \| \| \| \| \| \| \| --- \| --- \| --- \| --- \| --- \| --- \| --- \| --- \| --- \| --- \| --- \| --- \| --- \| \|  \|  \| **n** \|  \|  \|  \| **% (95%CI)** \|  \| **n** \|  \|  \|  \| **% (95%CI)** \| \|  \| **N, without missing** \| **Yes** \| **No** \|  \| **Missing** \| **Missing coded as “No”** \| **N, without missing** \| **Yes** \| **No** \|  \| **Missing** \| **Missing coded**  **as “No”** \| \| **Awareness** \|  \|  \|  \|  \|  \|  \|  \|  \|  \|  \|  \|  \| \| Flavor capsules \| 4056 \| 527 \| 3529 \|  \| 704 \| 11.1 (10.2, 2.0) \| 637 \| 125 \| 512 \|  \| 231 \| 14.4 (12.2, 16.9) \| \| Flavoring spray/drops \| 4036 \| 516 \| 3520 \|  \| 724 \| 10.8 (10.0, 11.7) \| 633 \| 103 \| 530 \|  \| 235 \| 11.9 (9.9, 14.9) \| \| Flavoring cards \| 4023 \| 293 \| 3730 \|  \| 737 \| 6.1 (5.5, 6.9) \| 630 \| 60 \| 570 \|  \| 238 \| 6.9 (5.4, 8.8) \| \|  \|  \|  \|  \|  \|  \|  \|  \|  \|  \|  \|  \|  \| \| **Ever use** \|  \|  \|  \|  \|  \|  \|  \|  \|  \|  \|  \|  \| \| Flavor capsules \| **-** \| **-** \| **-** \| **-** \|  \| **-** \| 637 \| 30 \| 607 \|  \| 231 \| 3.5 (2.4, 4.9) \| \| Flavoring spray/drops \| **-** \| **-** \| **-** \| **-** \|  \| **-** \| 632 \| 22 \| 610 \|  \| 236 \| 2.5 (1.7, 3.8) \| \| Flavoring cards \| **-** \| **-** \| **-** \| **-** \|  \| **-** \| 630 \| 17 \| 613 \|  \| 238 \| 2.0 (1.2, 3.2) \| |
| --- | --- | --- | --- | --- | --- | --- | --- | --- | --- | --- | --- | --- | --- | --- | --- | --- | --- | --- | --- | --- | --- | --- | --- | --- | --- | --- | --- | --- | --- | --- | --- | --- | --- | --- | --- | --- | --- | --- | --- | --- | --- | --- | --- | --- | --- | --- | --- | --- | --- | --- | --- | --- | --- | --- | --- | --- | --- | --- | --- | --- | --- | --- | --- | --- | --- | --- | --- | --- | --- | --- | --- | --- | --- | --- | --- | --- | --- | --- | --- | --- | --- | --- | --- | --- | --- | --- | --- | --- | --- | --- | --- | --- | --- | --- | --- | --- | --- | --- | --- | --- | --- | --- | --- | --- | --- | --- | --- | --- | --- | --- | --- | --- | --- | --- | --- | --- | --- | --- | --- | --- | --- | --- | --- | --- | --- | --- | --- | --- | --- | --- | --- | --- | --- | --- | --- | --- | --- | --- | --- | --- | --- | --- | --- | --- | --- | --- | --- | --- | --- | --- | --- | --- | --- | --- | --- | --- |
